# Themes in Abortion Forum Discussions in a Restrictive Access Context: Qualitative and Quantitative Analyses

**DOI:** 10.2196/59544

**Published:** 2024-12-09

**Authors:** Krzysztof Bartosz Klimiuk, Amelia Kot, Ewa Majcherek, Katarzyna B Kubiak, Łukasz Balwicki

**Affiliations:** 1 Department of Public Health and Social Medicine Faculty of Health Sciences Medical University of Gdańsk Gdańsk Poland; 2 Faculty of Medicine Medical University of Gdańsk Gdańsk Poland; 3 Faculty of Medicine Poznan University of Medical Sciences Poznań Poland

**Keywords:** abortion, content analysis, reproductive health, online forum, Poland, women, support

## Abstract

**Background:**

Abortion is one of the most common medical procedures worldwide. Despite this, access to abortion on demand remains restricted in many countries, including Poland. As a result, many women resort to undergoing the procedure without medical supervision, putting themselves at risk of serious health consequences such as drug poisoning, excessive bleeding, and hypovolemia. Unfortunately, some abortions also lead to severe infections.

**Objective:**

This study aims to qualitatively identify key themes in abortion forums to analyze the issues faced by individuals. The forums were then quantitatively analyzed to determine which problems were most prevalent.

**Methods:**

The most popular abortion forums were identified. A preliminary thematic analysis was conducted for the qualitative phase, followed by a manual quantitative analysis. Two independent researchers reviewed forum posts, categorizing them into specific themes.

**Results:**

An analysis of 13,397 responses from 370 threads across 4 forums revealed “Abortion Process Progression” as the most discussed theme, highlighting a strong demand for information, emotional support, and medical guidance. The highest percentage of threads (162/886, 18.3%) focused on the need for mental support and the desire to have someone in contact during the abortion process. Concerns about the effectiveness of the termination also emerged as a significant topic, accounting for 83 of the 886 (9.4%) discussions. “Emotional and Psychological Aspects” and “Medical and Pharmacological Aspects” were also significant, highlighting the need for holistic care. Anxiety and fear related to the process were frequently discussed, accounting for 60 of the 886 (6.8%) responses. The issue of isolation and lack of support was also a common topic, appearing in 30 of the 886 (3.4%) threads. Notably, “Social and Ethical Perception” accounted for only 13 of the 886 (1.5%) responses and appeared in just 13 threads.

**Conclusions:**

This study highlights the critical need for information and support for women navigating abortion, particularly in regions where access is restricted. It emphasizes the importance of addressing the multifaceted challenges women face and calls for policy changes and strengthened support networks to improve the health and rights of women, as well as all those seeking gynecological care in abortion contexts. Further research is encouraged to refine and expand support strategies.

## Introduction

Numerous countries, including Poland, are changing their laws to tighten access to abortion [1]. International discourse increasingly suggests that abortion should be viewed not just as a health issue but fundamentally as a human right [2]. Notably, the 1994 Conference on Population and Development in Cairo established a cohesive approach to population policies worldwide and recognized the rights of women to access abortion and contraception. The Cairo Action Plan was designed to allow its principles to be adapted to the national laws of each country, ensuring that the diverse religious, cultural, and ethical contexts of different nations were respected and upheld [3].

Limited access to abortion, or its absence, poses a significant challenge to public health [4]. Abortion is among the primary causes of maternal mortality. A study conducted across 115 countries between 2003 and 2009 documented that 7.9% of maternal deaths were attributable to abortion [5]. The actual number of deaths resulting from abortion could be higher due to potential underreporting [6]. Paradoxically, legalizing abortion can help reduce its incidence, as women receive improved postabortion care and access to contraception consultations [7].

The abortion law in Poland has experienced significant fluctuations over the decades, reflecting changes in the country’s political landscape [1,8]. Since 1997, the so-called abortion compromise—a middle ground between those advocating for liberalized abortion laws and those supporting a total ban—has allowed the procedure under 3 specific circumstances [9]. However, recent years have seen mounting pressure from antiabortion movements and influential segments of the Catholic Church [10], which holds significant sway in Polish politics. In 2020, the ruling party, Law and Justice (PiS), known for its staunch antiabortion stance, sought to introduce even stricter regulations [10,11]. That year, the Constitutional Court ruled that irreversible damage threatening the life of the fetus is not a prerequisite for terminating pregnancies under the Constitution, significantly restricting abortion rights in the country.

Since then, in Poland, pregnancy termination is legally permitted only in 2 scenarios: (1) when the pregnancy poses a threat to the life or health of the pregnant woman, or (2) when there is a reasonable suspicion that the pregnancy resulted from a prohibited act, such as rape [12,13].

An additional challenge is the right of conscientious objection, which allows any medical doctor to refuse to perform an abortion [14]. This has resulted in situations highlighted by the Commissioner for Human Rights in Poland, showing that in some regions, up to 1.1 million women lack access to abortion under any circumstances [15,16]. Consequently, women in Poland must seek alternative solutions, regardless of legal provisions [1,17].

Currently, there is no legal, official way to obtain an abortion in Poland outside of the specified indications. However, in the first trimester, a pharmacological termination of pregnancy is possible, with medications provided by international third-sector organizations [18]. This method, using Misoprostol, is one of the approaches recommended by the World Health Organization (WHO) [19].

Against the backdrop of evolving regulations in Poland, a country with one of the most restrictive abortion laws in the European Union [20], the authors have identified a gap in the literature regarding current studies on the situation of women who seek abortions outside official or legal facilities. The primary objective of this study is to qualitatively identify the challenges faced by women in Poland who wish to terminate their pregnancies. An attempt will also be made to approach these findings from a quantitative perspective to identify the most pressing public health challenges. In conclusion, these issues will be discussed in the context of current guidelines.

## Methods

### Study Overview

This observational study aimed to collect and analyze comments from online forums discussing abortion. Comments were gathered by reviewing various forums based on predefined criteria.

### Data Collection

Data collection and analysis were conducted from November 11, 2023, to December 13, 2023. The data were collected from the first page of Google (Alphabet Inc.) search results for the term “forum aborcja” (abortion forum). All forums on the first page containing discussions on abortion were included in the study. The data set covered posts from January 2023 to June 2023. Forum comments were categorized based on specific themes and subjects related to abortion discourse. To minimize bias and ensure reliability, 2 authors (KBK and AK) independently analyzed each comment. Any discrepancies were resolved through discussion. The analysis was supported by a spreadsheet documenting the source of each comment, its thematic classification, and relevant criteria based on the study’s categorization framework.

### Analysis Criteria

**Figure 1:**
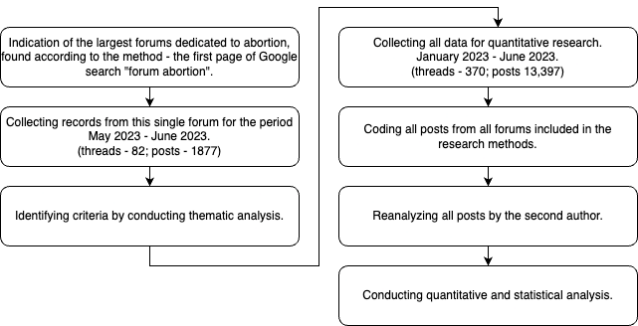
This flowchart illustrates the study's methodology, starting with identifying the largest abortion forum via Google search, collecting posts from May to June 2023 (82 threads, 1,877 posts), followed by coding, reanalysis, and quantitative analysis of data from all forums (370 threads, 13,397 posts) collected from January to June 2023.

A thematic analysis was conducted in July 2023 on forum threads from May and June 2023, following guidelines for thematic analysis [21]. The forum responses were analyzed using qualitative content analysis, based on the framework by Georgsson et al [22] and supplemented by the study of Aiken et al [23]. The manually collected responses were initially reviewed by the author KBK and refined by removing criteria that did not align with the research objectives. The criteria from the referenced studies were expanded to include additional factors based on themes emerging from the forum responses. The process of coding, data collection, and preparation is illustrated in Figure 1.

### Data Analysis

The data for this part of the analysis were collected from January to June 2023 and analyzed using a spreadsheet. The paper presents the quantitative occurrences of various themes. As multiple subcategories could appear within a single forum thread, the quantitative occurrences of entire categories were not aggregated. An analysis of the interdependence between subcategories was not conducted due to insufficient justification for such an approach.

### Statistical Analysis

A chi-square test was conducted to assess the relationship between the day of the week and the themes of forum threads. Additionally, another chi-square test was used to examine which themes cooccurred within threads. A significance level of .05 was applied, and the analysis was performed using R version 4.4.0 and RStudio version 2023.06.0 (R Foundation).

### Ethical Considerations

This observational study involved collecting and analyzing publicly available comments from online abortion forums, focusing on public behavior without direct interaction with participants. The data used were either anonymous or deidentified to ensure that no personally identifiable information could be traced to individuals. As all forum content was publicly accessible, formal ethical approval was not required. However, the authors adhered to ethical guidelines for internet research [24], ensuring user privacy by not disclosing any information that could identify individuals.

## Results

### Overview

A total of 13,397 responses from 370 threads across 4 forums were analyzed. A Google search (Google LLC/Alphabet Inc.) for the term “forum aborcja” (abortion forum) yielded 7 results, of which only 4 were actual forums. One link redirected to one of the mentioned forums, while the other 2 were articles about abortion. The search results and the number of threads collected are presented in Table 1.

**Table 1:** Categorization of the top search results from Google when querying “forum aborcja” at the beginning of the study^a^.

Source	Type	Number of forum threads analyzed
Aborcyjny Dream Team [25]	Redirects to Kobiety w Sieci [26]	0
Wolna Aborcja [27]	Forum	1
WP Parenting [28]	Forum	36
Kobiety w Sieci [26]	Forum	6
ABORCJA [29]	Forum	327
WP abcZdrowie [30]	Article	0
Aborcja Forum [31]	Prolife website with content discouraging the termination of pregnancy	0

^a^The entries are listed in the order they appeared in the search results. Each source is classified as a forum, article, or other type, and the number of forum threads analyzed from each source is indicated. Entries with no analyzed threads were excluded from further study.

### Analyzed Themes

#### Categorization and Analysis of Forum Threads

The collected and analyzed threads were categorized according to established criteria, which are detailed in Table 2. Each criterion was further divided into subcriteria to illustrate the prevalent themes. Table 2 shows the number of forum threads in which each theme appeared. Although a single thread could contain multiple themes, each specific theme within a given thread was counted only once, even if mentioned in multiple posts.

**Table 2:** Criteria for analyzing themes and their frequency in forum threads (N=886)^a^.

Themes and subthemes			Threads in which the theme appeared, n (%)
**Abortion Process Progression**			
	Questions and Description of Termination Process	162 (18.3)	
	Uncertainty About Procedure Effectiveness	83 (9.4)	
	Method of Medication Administration and Dosage	75 (8.5)	
	Alarming Symptoms and Complications	61 (6.9)	
	Failed Pharmacological Abortion	16 (1.8)	
**Emotional and Psychological Aspects**			
	Anxiety Related to the Process	60 (6.8)	
	Lack of Support and Isolation	30 (3.4)	
	Impact on Mental Health and Well-Being	19 (2.1)	
**Medical and Pharmacological Aspects**			
	Pharmacological Interactions (eg, medications taken, alcohol, tobacco)	28 (3.2)	
	Impact on Health With Preexisting Conditions	18 (2.0)	
	Postabortion Contraception Options	18 (2.0)	
	Long-Term Side Effects	17 (1.9)	
**Logistical Issues and Specific Situations**			
	Long Wait for Medication	54 (6.1)	
	Challenges in Caring for Existing Children	33 (3.7)	
	Hiding Procedure From Family Or Partner	30 (3.4)	
	Uncertainty About Pregnancy Presence or Gestational Week	29 (3.3)	
	Alternative Methods of Termination	18 (2.0)	
	Logistical and Financial Barriers	13 (1.5)	
	Concerns About Postprocedure Activities (eg, returning to work)	10 (1.1)	
**Issues Related to Assistance From Medical Staff**			
	Doctor’s Ability to Identify an Abortion	21 (2.4)	
	Concerns About Medical Procedures (eg, uterine curettage)	15 (1.7)	
	Recording Pregnancy in the Medical System	14 (1.6)	
	Fear of Medical Staff’s Reaction	9 (1.0)	
**Legal and Ethical Issues**			
	Online Scams and False Offers	19 (2.1)	
	Social and Ethical Perception	13 (1.5)	
	Legal Consequences of Abortion	11 (1.2)	
	Legislation and Restrictions	10 (1.1)	

^a^Each theme is broken down into subcriteria to indicate how often it appeared across all threads. Although a single thread could include multiple themes, each theme was counted only once per thread, even if mentioned in several posts.

#### Abortion Process Progression

##### Overview

The findings in the “Abortion Process Progression” category provide valuable insights into forum discussions about abortion. They highlight a strong need for information and support on various aspects of the process, including practical advice on medication use and emotional assistance for complications and uncertainties.

##### Questions and Description of Termination Process

This was the most discussed theme, appearing in 162 of the 886 (18.3%) threads, indicating a significant demand for information and clarity about the abortion process. Discussions likely included questions about the procedure itself, what to expect, and personal experiences shared by those who had undergone it. This criterion included threads where people provided real-time updates on what was happening, as well as threads where individuals shared detailed personal experiences.

Hey, maybe it’s because women are somehow embarrassed to write about what happens to their bodies during such a procedure. For me, nothing human is alien, so after the first dose, I had mega chills, diarrhea, and I felt nauseous. After the second dose, my stomach hurt a lot, and after the third dose, the chills and diarrhea stopped, but I started having terrible cramps :/ and such strong stomach pain that I had to use painkillers, otherwise, I don’t know if I would have managed :/ I expelled a clot about a third of the size of my palm with a membrane...so I suspect that was it. After that, the pain decreased, and today my bleeding is like during a normal period. I still pass small clots, so as I understand, the uterus is cleaning itself, but besides that, I feel okay. I have an ultrasound scheduled, I will go and check if everything is okay, but yes, I feel that it went successfully.

##### Uncertainty About Procedure Effectiveness

Doubts regarding the effectiveness of the abortion procedure were discussed in 83 of the 886 (9.4%) threads. These discussions likely included concerns about confirming the procedure’s success, such as monitoring human chorionic gonadotropin (hCG) levels, highlighting anxiety, and the need for reassurance about the outcomes.

Hey girls, listen, I started the treatment on Friday. After the first dose, I had a slight stomach ache, after the second one, diarrhea and stomach pain, and after the third, there was spotting and 2 or 3 small clots, about 2 cm, came out. The next day there was only light brown spotting. This is the 5th week. I’ll be honest, I don’t know if it was successful, I thought maybe something would develop more after 3 days, but nothing. I’ll also add that I didn’t have the typical bleeding that the girls here describe, I went through it very mildly. Please support me.

##### Method of Medication Administration and Dosage

This theme appeared in 75 of the 886 (8.5%) threads, indicating notable concern about the practical aspects of medication-based abortions. Discussions likely focused on the correct use of abortion pills, dosage instructions, and handling issues such as a crushed tablet, highlighting the need for accurate medical guidance. Questions included methods of drug administration and dosing details.

Has anyone taken the pills vaginally? Supposedly, they are more effective this way?

Hello girls, today I took the first pill and I have a question: if I took it at 15:00, can I start two hours earlier tomorrow?

As well as more specific situations:

Girls, in the package, a few pills were open, and this one big pill was crushed. What now? Will the big one still work even though it’s crushed? Help please.

##### Alarming Symptoms and Complications

This criterion, appearing in 61 of the 886 (6.9%) forum discussions, was identified when users reported serious side effects of the medications and the abortion procedure, both during and after, extending up to the next menstrual cycle. It included descriptions of intense or persistent bleeding and pain, as well as excessive diarrhea, vomiting, and other atypical symptoms that could pose health risks.

Girls, are you also bleeding so heavily? I’ve been bleeding non-stop since Monday, and my stomach hurts, is this normal?

I once had an allergic reaction to the medication; if such a reaction starts affecting your décolletage, neck, feet, etc., this is serious, and I advise going to the emergency room. [...] If it gets worse, what to tell the doctor? Nothing...just show your skin, that’s enough.

I can’t deal with the pain; it’s terrible. I don’t know if natural childbirth hurt as much as it does now, I’m writhing in pain on the bed, one cramp after another like crazy. There’s no bleeding. The diarrhea was severe but now it’s somewhat okay. The last dose is at 3 and I can barely function...It’s just terrible

##### Failed Pharmacological Abortion

The least discussed but still notable, this theme appeared in 16 of the 886 (1.8%) threads. It indicates that while less common, there are cases where pharmacological abortions fail, prompting discussions on how to identify failure and what actions to take next.

Girls, unfortunately, it didn’t work 😔 Yesterday I ordered another set, and I’m starting again next week, it’s a nightmare.

#### Emotional and Psychological Aspects

##### Overview

Findings within the “Emotional and Psychological Aspects” category reveal that the emotional and psychological components of abortion are a crucial part of the experience, highlighting a clear need for support systems that address these issues. The data emphasize the importance of providing comprehensive care that includes psychological support for individuals undergoing an abortion.

##### Anxiety Related to the Process

Anxiety was a notable concern, with 60 of the 886 (6.8%) threads reflecting apprehension about the process. Many users expressed uncertainty about what to expect, the severity of pain, and the duration of the procedure. This anxiety could stem from the fear of the unknown, worries about potential complications, or stress related to the decision itself.

Girls, I’m really scared. I’m in the 14th week. It turns out that my son has defects. I can’t afford to travel abroad, and in Poland, you know - it may or may not work. That’s why I want to try with the pills. I’m just wondering whether A) it’s too late B) maybe I should order two sets right away. Did any of you have a situation where the child survived the treatment? Or did anyone do it this late and it was successful?

##### Lack of Support and Isolation

The issue of feeling unsupported and isolated was present in 30 of the 886 (3.4%) discussions, highlighting the emotional difficulty some individuals face when lacking a supportive network during the abortion process. This can exacerbate the emotional distress associated with the experience. In some cases, forum members were the only people aware of the user’s abortion and the only source with whom they could discuss their concerns and the process.

oh that’s nice, I don’t have such a doctor. I’m terribly afraid of this treatment and I’ll also be alone at home and I have anemia as well.

##### Impact on Mental Health and Well-Being

The impact on mental health and well-being, though less frequently discussed in 19 of the 886 (2.1%) threads, was still notable. This subcategory encompassed discussions on emotional responses following an abortion, including feelings of relief or regret, and the overall psychological impact of the procedure.

I guess the strongest thing I feel is emptiness and the awareness of loss. I need to somehow sort this out in my head. To go home, tell my 10-year-old, who is waiting for me, that the baby is no more. Then, to return to work, where everyone knew about the pregnancy.

I wonder if it would be a good idea to ask one of my colleagues to inform the rest about my situation, so that no one would ask me how I and the child are feeling.

#### Medical and Pharmacological Aspects

##### Overview

The results in the “Medical and Pharmacological Aspects” category highlight the critical need for comprehensive medical information and guidance for individuals considering or undergoing an abortion. This includes managing long-term health, understanding drug interactions, evaluating preexisting conditions, and planning for postabortion contraception.

##### Pharmacological Interactions (eg, Medications Taken, Alcohol, Tobacco)

This theme, accounting for 28 of the 886 (3.2%) threads, highlights concerns about potential interactions between abortion pills and other medications, as well as alcohol or tobacco use.

The most frequently asked questions in this thread revolved around alcohol and tobacco consumption. Members wanted to know if it was safe to drink alcohol during the procedure, hoping it would help ease their stress. Discussions also included concerns about the use of medications for current and chronic conditions, with members expressing fears about potential drug interactions and whether abortion medications would be effective when combined with other substances.

Hey, does anyone know if taking antibiotics will interfere with the abortion procedure? I was prescribed antibiotics by the dentist today for a tooth infection, and I don’t know if I can take them...

And tell me, darling, when can I smoke? I’m so nervous that a cigarette could save me.

##### Impact on Health With Preexisting Conditions

Representing 18 of the 886 (2.0%) threads, this topic highlights concerns about how undergoing an abortion might impact individuals with preexisting health conditions. These discussions often included worries about potential complications during the procedure or the possibility that it could worsen existing health issues.

For me, it was a nightmare!:/ I was completely exhausted. The bleeding lasted very long, plus I had a tense stomach :/ and I only thought about being active again after 2 months. after a month???

##### Postabortion Contraception Options

Making up 18 of the 886 (2.0%) threads, this subcategory indicates that participants were seeking information and advice on contraception following an abortion. Forum members questioned whether hormonal contraception could be used immediately after an abortion and were curious about the contraindications of various contraceptive methods post-abortion.

Hey, I’m looking for a clinic/doctor in Germany who will perform a tubal ligation/sterilization procedure. I live near the border (Lubuskie). Unfortunately, I don’t speak the language to call around and ask.

##### Long-Term Side Effects

This subcategory, comprising 17 of the 886 (1.9%) discussions, involved conversations about potential long-term side effects following the abortion procedure. Some participants expressed concerns about future fertility, fearing that they might have difficulty getting pregnant after an abortion. Additionally, there were questions about whether any tests or treatments were necessary after undergoing the procedure.

Hello everyone, I was reading on IG lekarkiproabo about serologic conflict, precisely, “Many people plan to have children in the future after an abortion, so it’s important to know your blood type and, if necessary, see a doctor for prophylaxis.” I hadn’t heard about this before, and I’ve already had three abortions. I don’t know my blood type; I’m going to get it tested tomorrow. I want to have children in the future. Presumably, if it turns out I am Rh-, does the fact that I never received/didn’t go for a prophylactic injection after the abortions mean I might have problems in future pregnancies? I read that it can cause miscarriages and generally not good things...:( Request for clarification.

#### Logistical Issues and Specific Situations

##### Overview

The results in the “Logistical Issues and Specific Situations” category highlight the diverse and complex logistical challenges faced by individuals considering or undergoing an abortion. This underscores the need for accessible, supportive, and comprehensive abortion care services.

I can’t go out to get a test because I have sick children at home and no one to leave them with. And I don’t want to ask anyone to buy it for me because I don’t want anyone to know.

##### Long Wait for Medication

A notable concern, discussed in 54 of the 886 (6.1%) threads, was the prolonged waiting period to receive abortion medication. This delay heightened stress and anxiety for forum members, who were worried that their ordered medication might arrive after the 9th week of pregnancy, when pharmacological abortion would no longer be an option. Consequently, some members ordered multiple sets from different sources simultaneously, and others even considered buying pills from strangers on the forum, an action that is illegal in Poland.

Hey. I ordered a set two days ago. I’m currently 5 weeks along. The order was confirmed yesterday, but the package has not yet been dispatched. My mind is in turmoil. [...] I’m from a small town in Podlasie, and if this got out, I would be lynched. I just don’t know what to do with myself. It’s nice that you are here, and I really count on your support :)

##### Challenges in Caring for Existing Children

Accounting for 33 of the 886 (3.7%) discussions, this theme highlights the difficulties faced by individuals with children who are considering or undergoing an abortion. Balancing childcare responsibilities during this process poses unique challenges. Many mothers shared that they planned to have an abortion when their children were in kindergarten, school, or during the night while they were asleep. Forum members expressed concerns about their ability to care for their children during the procedure. Additionally, some individuals worried about their capacity to support another child financially and emotionally, which influenced their decision to undergo an abortion.

##### Hiding Procedure From Family or Partner

This subcategory, present in 30 of the 886 (3.4%) threads, highlights that many individuals felt the need to hide their abortion from family members or partners, reflecting the social stigma and personal conflict associated with the decision. Reasons for concealing the procedure varied across the threads. For example, some individuals were in abusive relationships and feared for the future of their children, while others faced differing opinions about having children within their relationships. When it came to hiding the abortion from family members (eg, mom and dad), religious beliefs and faith were often cited as the primary reasons for maintaining secrecy.

Hey girls. I’ve decided to have an abortion. I’m at the beginning of the 5th week. I told my husband initially that I wanted an abortion because we are in a difficult situation; we live separately, he’s abroad, and this won’t change any time soon. But now I’m wondering whether to tell him that I’m going to do it, or just blame it on nature, I don’t know if he’ll be able to handle it psychologically. I’m just waiting for the pills. I live with my parents and have to keep all of this a secret.

##### Uncertainty About Pregnancy Presence or Gestational Week

This subcategory, making up 29 of the 886 (3.3%) discussions, highlights the uncertainty that some forum participants had regarding their pregnancy status or the specific week of their pregnancy. Forum members frequently asked for help interpreting beta-hCG test results. This uncertainty can notably influence the decision-making process and the choice of the abortion method.

Hey girls. I’m totally new here, reading your threads, and it’s easing my stress a bit, but I’m still loaded with emotions...[...] The worst part is that I can’t remember when I last had my period. I stopped taking birth control pills about 3 or 4 weeks ago, that’s when I had my period, but unfortunately, I didn’t note the exact date...[...] On Friday, I did two pregnancy tests, they came out positive. For me, it was a shock because I totally didn’t expect it, I had my period just a short while ago...I have no idea when exactly the fertilization could have happened, so I don’t even know how to calculate it :( Today I went for a beta test, tomorrow I’m picking up the results to be 100% sure and to figure out approximately which week it will be.

##### Alternative Methods of Termination

Present in 18 of the 886 (2.0%) discussions, this topic reflects the exploration or pursuit of alternative methods for abortion, possibly due to the inaccessibility or unavailability of preferred options. Forum members inquired about what the procedure would be like in abortion clinics abroad, its cost, the quality of care, and whether they could communicate effectively with doctors from another country.

Girls, I have an issue. I’ve been through two unsuccessful treatments. Now I’m looking for a reliable clinic. Has any of you had any experience with clinics abroad?? If so, please help me with some verified contact information. There’s a lot on the internet but you never know where you might end up.

##### Logistical and Financial Barriers

Making up 13 of the 886 (1.5%) discussions, this theme highlights the logistical and financial obstacles encountered in accessing abortion services. These barriers can include travel costs, medical fees, and the complexity of arranging the procedure. Additionally, people living in rural areas expressed concerns that their medication parcels might not arrive on time, and some were worried that if complications arose, they would be unable to reach a hospital for professional help.

Girls, I’m really scared. I’m in the 14th week. It turns out that my son has defects. I can’t afford to travel abroad, and in Poland, you know - it may or may not work. That’s why I want to try with the pills. I’m just wondering whether A) it’s too late B) maybe I should order two sets right away. Did any of you have a situation where the child survived the treatment? Or did anyone do it this late and it was successful?

##### Concerns About Postprocedure Activities (eg, Returning to Work)

Making up 10 of the 886 (1.1%) threads, concerns about postprocedure activities were prevalent. Forum members were uncertain about the recovery period and the duration of bleeding, which could impact their return to work. Additionally, there were questions about how soon after the abortion it would be safe to have sexual intercourse with a partner.

How long after taking the pills can I return to full physical activity?? I mean doing everything like work, sports, etc? I’m a professional swimmer and I wouldn’t want this to negatively affect my body.

#### Issues Related to Assistance From Medical Staff

##### Overview

The results under the category “Issues Related to Assistance From Medical Staff” reveal the challenges forum participants faced when interacting with medical professionals during the abortion process. These findings highlight the complexities and anxieties surrounding medical staff interactions and underscore the need for sensitive, nonjudgmental, and supportive care for individuals undergoing an abortion.

##### Doctor’s Ability to Identify an Abortion

Accounting for 21 of the 886 (2.4%) threads, this subcategory reflects concerns about whether doctors can differentiate between a spontaneous miscarriage and an induced abortion. Additionally, threads included questions about when during a medical abortion participants should go to the hospital, believing the pregnancy could no longer be saved. The topic also covered whether gynecologists could identify that a miscarriage was induced and if doctors asked questions to confirm this.

I have a question, girls, If I go through with the procedure on Monday, and I have a gynecologist appointment on Wednesday, is there a possibility that the gynecologist will realize that it wasn’t a spontaneous miscarriage?

##### Concerns About Medical Procedures (eg, Uterine Curettage)

Making up 15 of the 886 (1.7%) threads, this theme highlights fears surrounding the medical procedures associated with abortion, such as uterine curettage. Participants expressed anxiety about whether they would be given an injection and sent home immediately or if they would need to stay for further tests and procedures. There was particular concern about the standard practice of curettage in their region. Additionally, forum members questioned the necessity of using curettage. Many expressed the belief that curettage was unnecessary, as they thought the uterus would naturally “clean itself” after an abortion.

Will they give me the injection right away and send me home? Or will they want to keep me for more tests? An ultrasound? That dreaded curettage, which, at least in my region, seems to be the standard...That’s what I’m most afraid of.

##### Recording Pregnancy in the Medical System

Representing 14 of the 886 (1.6%) discussions, this topic highlights concerns regarding the recording of medical details, such as beta-hCG test results, in medical registries. Participants expressed worries about the privacy and confidentiality of their medical information related to pregnancy and abortion. Many were anxious that, after a positive beta-hCG result, a pregnancy card would be added to their medical records, and if they underwent an induced miscarriage, they would need to explain the circumstances of the miscarriage.

Girls, I have a question, do you know if beta test results are entered into a registry?

##### Fear of Medical Staff’s Reaction

This subcategory, making up 19 of the 886 (1.0%) discussions, reflects apprehension about how medical staff might react upon learning about an abortion or pregnancy. Forum members shared concerns and negative experiences involving judgmental attitudes from health care providers. For instance, some participants recounted instances where doctors made critical or biased remarks, such as reprimanding them for being pregnant at a young age or assuming they would be single mothers, which added to their anxiety and feelings of discomfort during the medical process.

I’ll describe how the entire visit went. I arrived and said that I mainly wanted an ultrasound because I suspected I was pregnant. From the beginning, he was unfriendly to me, making comments like “at such an age and already pregnant” (I’m 20 years old this year). When I said I was in the 14th week, I burst into tears out of nerves because I thought it was too late for an abortion, but I knew I couldn’t mention anything about it. After the examination, he started asking if I had a partner; I briefly answered that he’s abroad. He then started saying that in that case, he must be a bad partner, that I am therefore a single mother, and that I need to think about my actions. I didn’t respond at all, just tears were streaming down my face from stress. He said that there’s a place for people like me and then he wrote down the number of this home for single mothers XD. He made up the whole narrative while being a complete jerk, while I didn’t say a word about whether I want or don’t want the child. He asked me if I wanted him to manage my pregnancy; I said I didn’t know because I’m in shock and need to think about it. After that answer, he snorted, Well well, it’s your decision. That’s all, goodbye.

#### Legal and Ethical Issues

##### Overview

The findings in the “Legal and Ethical Issues” category highlight the multifaceted concerns and experiences shared by forum participants, illustrating the challenges related to the legal, ethical, and social aspects of abortion. The discussions revealed apprehensions about potential legal repercussions, the fear of being reported, and the ethical dilemmas that come with seeking abortions outside of official channels. The conversations emphasized the importance of creating supportive and secure spaces for individuals to discuss their situations without fear of judgment or legal consequences.

##### Online Scams and False Offers

Representing 19 of the 886 (2.1%) discussions, this theme involves experiences of being scammed while attempting to obtain abortion pills online. Users in this thread also debated whether the offers found on the internet were fraudulent posts made by the Polish police or by individuals associated with the government. One user shared:

I came here on a friend’s recommendation, just finished the treatment, and before that, I was scammed twice! I naively transferred the money and saw nothing :/ I can’t understand how someone can behave like this towards other people!

##### Social and Ethical Perception

Making up 13 of the 886 (1.5%) threads, this subcategory reflects the social and ethical attitudes and emotions surrounding abortion. Some of the most fearful users were those from small communities, concerned that their neighbors would discover their abortion and ostracize them. These threads also included users grappling with guilt following their abortion. One user asked:

Hey, I’d like you to share how you felt psychologically a few months...a few years after the abortion? Did any memories, guilt come back?

Another harsh comment read:

All of you undergoing abortions should be locked up in a cage. Trash.

##### Legal Consequences of Abortion

Accounting for 11 of the 886 (1.2%) discussions, this topic covers fears and questions regarding the legal implications of purchasing abortion pills in Poland. There were numerous inquiries and concerns about the current abortion laws and the potential consequences for those who have undergone an abortion. Some users also questioned the exact definition of “assistance in having an abortion,” which is illegal in Poland. A forum participant expressed:

Many people warn me against buying pills in PL. They scare me with the police. How is it? I’m desperate because I need the pills to cleanse myself.. I need them fast. What are your experiences? Aren’t you afraid to order?

##### Legislation and Restrictions

This subcategory, making up 10 of the 886 (1.1%) discussions, involves concerns about the legal aspects and restrictions related to abortion. Although similar to the previous thread, this one included posts discussing direct contact with the justice system, such as being summoned to the police station for questioning. For example, one participant shared:

Hi girls, I haven’t been on the forum for a long time. Today, I’ve been a mother for 4 months, I have a son and I’m very happy although I won’t hide that I used to think that it wasn’t for me. To the point, did any of you recently receive a summons to the police as a witness? The articles cited in the summons are: 152 § 2 of the Penal Code - punishable assistance in abortion; 127a sec. 2 of the Pharmaceutical Law - mediating in the trade of medicinal products without the required entry or permission; 270 § 1 of the Penal Code - forging documents. It surely isn’t about you, I bought pills from wow, but before I learned about women on the internet, I once ordered pills in 2019 from the site 9tygodni.pl (through which I was later blackmailed to tell my family). Did any of you have such an interrogation? What to expect?

### Statistical Analysis

#### Day of the Week and Themes

There was no significant relationship between the day of the week and the themes discussed in the threads (chi-square test, *P*=.22). This indicates that the occurrence of themes was independent of the day of the week.

#### Occurrence of Threads

The theme of Abortion Process Progression was more frequently discussed in conjunction with Emotional and Psychological Aspects and Legal and Ethical Issues compared with other themes. Logistical Issues and Specific Situations were more commonly associated with Emotional and Psychological Aspects and Issues Related to Assistance From Medical Staff. Lastly, Legal and Ethical Issues were frequently discussed alongside Issues Related to Assistance From Medical Staff. The *P* values from the chi-square test are presented in Table 3.

**Table 3:** *P* values of the chi-square test to determine which themes occurred together in threads.

Themes	Abortion Process Progression	Emotional and Psychological Aspects	Medical and Pharmacological Aspects	Logistical Issues and Specific Situations	Issues Related to Assistance From Medical Staff	Legal and Ethical Issues
Abortion Process Progression	x^a^	.008	.23	.33	.42	<.001
Emotional and Psychological Aspects	.008	x	.56	<.001	.13	.29
Medical and Pharmacological Aspects	.23	.56	x	.82	.11	.33
Logistical Issues and Specific Situations	.33	<.001	.82	x	.01	.32
Issues Related to Assistance From Medical Staff	.42	.13	.11	.01	x	.001
Legal and Ethical Issues	.001	.29	.33	.32	.001	x

^a^This value was entered when the same category appears in both the row and column.

## Discussion

### Principal Findings

In response to the 2020 restrictions, which effectively implemented a near-total ban on abortion across the country, women seeking abortion care in Poland often rely on community support from specific organizations [12]. Additionally, they may organize trips abroad, purchase medications on the black market, or import them from other countries. However, there is a lack of an official, fact-based source of information developed by specialists. All necessary information must be obtained from forums, chats, and internet pages, some of which have been analyzed in this work [9,18].

Following the introduction of near-total abortion restrictions, the number of legal abortions in Poland decreased significantly. According to data from the Polish Ministry of Health, there were 1110 legal abortions in Poland in 2019 and 1076 in 2020. This number dropped 10-fold to 107 legal abortions in 2021 and slightly increased to 161 in 2022 [32]. Referring to data from major aid organizations, it is believed that the reported statistics significantly underestimate the actual number of abortions taking place in the country [33,34].

The Abortion Dream Team, the largest organization in Poland providing abortion assistance and uniting other aid organizations, supported 32,000 individuals in accessing abortion services in 2021 and facilitated travel for 1544 individuals to foreign abortion clinics. Between October 22, 2021, and October 17, 2022, the Abortion Dream Team assisted 44,000 individuals in obtaining abortions [34,35]. The Polish Foundation for Women and Family Planning (FEDERA), in the 2 years following the court ruling that restricted abortion access, provided services to thousands of women. This included handling over 20,000 helpline calls, responding to 12,000 emails, offering 1016 consultations for refugees from Ukraine, providing 1200 legal consultations, intervening in 28 hospitals, and filing 30 complaints to the European Court of Human Rights regarding hospital violations of patients’ rights [33].

Our analysis indicates that many individuals have sought abortion support independently through unofficial online forums, suggesting significant underestimation and underreporting of abortion statistics (a total of 13,397 responses from 370 threads across 4 forums were analyzed). This highlights the lack of reliable data and comprehensive discussions about abortion in Poland. Thousands of individuals who have undergone the procedure remain in a gray area, with their experiences unrepresented in official statistics.

This research aims to highlight the perspectives of those with firsthand experience. The objective was to gain insight into the decision-making process of individuals who choose to terminate pregnancies, exploring the broader context of their situation, as well as their concerns and obstacles. The results show that women face a range of challenges related to abortion, from technical and clinical aspects—such as selecting the appropriate medication, timing, and dosage—to the actual process of terminating the pregnancy, including how it should unfold and potential complications. Our analysis parallels the work of Georgsson et al [22], highlighting the urgent need for comprehensive abortion care that includes both procedural information and emotional support. Both studies reveal a significant gap in patient knowledge and preparation, emphasizing the importance of providing accessible, accurate information about abortion procedures. A shared focus on the emotional and psychosocial support required to address concerns about physical pain, procedure effectiveness, and the psychological aftermath further underscores the necessity of a holistic approach to care. Additionally, the prominence of online forums in our study aligns with Georgsson et al [22]’s recognition of digital platforms as essential resources for support, suggesting an integrated model of health care that combines both traditional and digital approaches to meet the diverse needs of individuals undergoing an abortion.

Inquiries about the lack of knowledge regarding the abortion process are the most common, accounting for 162 of the 886 (18.3%) forum discussions. These primarily focus on questions and descriptions of the termination process. Alarmingly, the issues raised today strongly resemble those identified after 1993, when abortion rights in Poland were severely restricted—particularly the removal of termination options based on the difficult life circumstances of the pregnant woman [1,36-38]. During that period, researchers observed a decline in public awareness about pregnancy termination and an increase in the use of underground abortion practices or seeking terminations abroad, which significantly compromised patient safety. This situation not only reflects a repetition of past mistakes in public health policy but also represents a regression that further exacerbates these issues.

Legally, the situation today mirrors past issues, further complicated by the fears faced by both patients and physicians. The antiabortion law has historically been more restrictive in practice than its statutory language suggests. There is evidence showing that women, despite being entitled to a legal abortion and having all the required documentation, were still denied access to the procedure [1,38]. According to our data, 53 of the 886 (6.0%) forum users are currently facing legal and ethical issues related to abortion. This underscores the persistent challenges and barriers within the legal framework that continue to affect access to abortion services.

Patients express apprehensions about the potential detection of abortions by medical staff and concerns regarding the registration of pregnancies—these issues appeared in 21 of the 886 (2.4%) and 14 of the 886 (1.6%) analyzed texts, respectively. Since October 1, 2022, the mandatory recording of pregnancies in the medical events registry has subjected patients to oppressive measures [39]. The legal framework surrounding the “Pregnancy Registry” exacerbates women’s feelings of anxiety and oppression. There is a palpable fear that the registry information could prompt inquiries into the reasons for not proceeding with childbirth, undermining trust in the doctor-patient relationship and compromising the confidentiality of medical data. This situation has notably disturbed patients, further eroding their sense of privacy and confidence in health care services [40].

Ideally, such a registry could serve as a valuable data source for analyzing maternal health realities, identifying potential threats, and enhancing guidelines and preventive measures, similar to practices applied to certain states or medical conditions [41-43]. However, in the current legal context, achieving these objectives seems unattainable without jeopardizing patients’ sense of security and legal safety.

Polish Public Health is responsible for ensuring abortion safety amid unfavorable legal and systemic conditions, making this study one of the few of its kind in Poland. Currently, there is a noticeable lack of official health data that highlight the challenges faced by individuals during abortions. Through this analysis, we are able to outline the problems, concerns, and aspects of the abortion process experienced by Polish women. As a result, it becomes possible to develop targeted support strategies, systemic solutions, and improve patient safety by providing accurate medical information. However, it is clear that more studies of this nature are urgently needed.

Comprehensive abortion care, recognized by the WHO as part of the List of Essential Health Services, requires strategic initiatives [44]. By conducting appropriate educational campaigns, decriminalizing abortion, and ensuring access to medical assistance, we could systematically address 23 out of the 27 issues identified in our study (Table 1). Information on both pharmacological and surgical abortion procedures should be made accessible to patients and health care professionals [45].

It is crucial to focus on providing information and support to marginalized groups, including low-income women and girls, refugees and migrants, youth, lesbians, bisexual cisgender women, transgender or nonbinary individuals, and members of ethnic and religious minorities [46,47]. This is especially important in light of the ongoing armed conflict in Ukraine, which has driven many women to migrate to Poland in search of reproductive health services, including abortion [48].

Patients should have the right to access abortion services within the Polish health care system, supported by doctors, midwives, or appropriately trained staff, and receive comprehensive pre- and postabortion care. The framework for such access suggests that decriminalization of both those seeking and providing abortions could make a significant contribution. Ensuring access to high-quality, evidence-based information could enhance patient safety in reproductive health, minimize health risks, and uphold fundamental human rights [49-51].

### Limitations

This research has several limitations. Primarily, as a content-based study, it faces typical constraints associated with this methodology, such as selection bias and limited generalizability, as it relies on data from individuals active in online communities [52]. This approach may not fully capture the diverse experiences of all women seeking abortions in Poland. Additionally, the study’s time frame (January to June 2023) limits the understanding of the evolving context of abortion access, which may affect the long-term relevance of the findings. The qualitative analysis introduces a level of subjectivity through the researchers’ interpretation. Another limitation is the possibility that forum moderators may have removed or edited posts, potentially affecting the data set’s completeness and the depth of the analysis. A sentiment analysis could have provided additional insights; however, due to the varying formats of the forums from which the data were collected and the differing terms of service, it was not conducted. Finally, systemic barriers within the health care system were not fully explored, highlighting an area for further research.

### Conclusions

This study is one of the first to explore the abortion experiences of Polish women in such comprehensive detail, shedding light on issues that often escape the attention of policy makers and health care practitioners. Through an in-depth analysis of online forum discussions, it uncovers the nuanced challenges women face under Poland’s restrictive abortion laws, highlighting the gap between legislative frameworks and the actual needs of women. The research highlights critical issues related to access, information, and emotional support—factors that are pivotal in shaping public health strategies and informing policy decisions. In doing so, it not only enriches the academic discourse on reproductive health but also signals to policy makers the urgent need for revisions that align with women’s rights and well-being.

Moreover, the findings highlight a stark contrast between Poland’s approach to abortion and the standards set by the European Union regarding women’s health care and rights. The study offers actionable recommendations for public health in Poland, including decriminalizing abortion, launching educational campaigns to combat misinformation, and strengthening support systems for women. These suggestions aim to bridge the gap between the current state of abortion services in Poland and the comprehensive care model advocated by international human rights standards, urging policy makers to guarantee women’s access to safe and supportive reproductive health care.

## Abbreviations

AI: artificial intelligence
FEDERA: Polish Foundation for Women and Family Planning
hCG: human chorionic gonadotropin
WHO: World Health Organization

## Data Availability

All data used in this study were publicly available at the time of collection. The forums and discussions analyzed are accessible through public internet sources. The combination and presentation of these data in the manuscript are considered exhaustive and comprehensive. Because of the public nature of the data, no additional data sets have been deposited in a separate repository. Any further details can be accessed directly from the sources mentioned in the manuscript.
